# The quintessential form of diastolic heart failure in older adults: Wild type transthyretin cardiac amyloidosis

**DOI:** 10.1002/clc.23301

**Published:** 2019-12-11

**Authors:** Elissa Driggin, Mathew S. Maurer

**Affiliations:** ^1^ Department of Medicine Columbia University Irving Medical Center New York New York; ^2^ Division of Cardiology, Department of Medicine Columbia University Irving Medical Center New York New York

**Keywords:** amyloid, heart failure, transthyretin

## Abstract

Wild‐type transthyretin cardiac amyloidosis (ATTRwt) is now recognized as a common cause of heart failure with preserved ejection fraction (HFpEF). In this review, we aim to describe the unique epidemiologic, pathophysiologic, and clinical features associated with ATTwt cardiac amyloidosis. Compared to other etiologies of HFpEF, ATTRwt cardiac amyloidosis affects almost exclusively older adults, demonstrating a characteristic age‐dependent penetrance that impacts both the diagnosis and treatment of the disease. In addition, ATTR cardiac amyloidosis demonstrates a unique pathophysiology in contrast to other etiologies of HFpEF, which results in a characteristic phenotype that can raise suspicion for ATTRwt cardiac amyloid in the appropriate demographic. With these distinguishing features in mind, we aim to describe the specific signs, symptoms, and imaging characteristics associated with ATTRwt cardiac amyloidosis, including the role of nuclear scintigraphy that has essentially eliminated the need for biopsy in most patients with suspected disease. Finally, we review the evidence behind the available therapeutic agents, as well as those under investigation, which will change the way we manage older patients with ATTRwt cardiac amyloidosis in the coming years.

## 1 INTRODUCTION

Among subjects with heart failure (HF), more than half have a preserved ejection fraction (HFpEF).^1^ The clinical syndrome of HFpEF encompasses a heterogeneous group of disorders with diverse pathophysiology and thus far treatment strategies to impact survival and quality of life have demonstrated limited success to date. Recently, wild type transthyretin cardiac amyloidosis (ATTRwt) has emerged as an exception. Previously thought of as a rare disease, ATTRwt cardiac amyloidosis is now recognized as an underdiagnosed cause of HFpEF that almost exclusively affects older adults, demonstrating a unique age‐dependent penetrance. As a result of the elucidation of the pathophysiology of ATTR cardiac amyloidosis, there has been translational triumph marked by advancements in both diagnosis and treatment of ATTRwt cardiac amyloidosis that will lead to meaningful changes in clinical outcomes including morbidity, mortality as well as functional capacity and quality of life, which is important to consider given the advanced age demographic that is affected.^2^ In this review, we will highlight important and current concepts in the epidemiology, pathophysiology, diagnosis, and treatment of ATTRwt cardiac amyloidosis in the older adult.

## 2 EPIDEMIOLOGY

ATTRwt cardiac amyloidosis is an underdiagnosed cause of HF in older adults. Some of the earliest evidence for the high prevalence of cardiac amyloid deposition in advanced aged populations comes from autopsy studies. For example, in a cohort of 85 patients over 80 years of age evaluated for systemic amyloid deposition at autopsy, 25% of myocardial specimens demonstrated cardiac amyloid deposition.^3^ Another study of 256 autopsy specimens from patients over 85 years of age identified a similar 25% prevalence of myocardial amyloid deposition.^4^ While both studies suggest high histologic prevalence, the clinical significance of these findings, specifically their relation to prevalence and incident HF was not clear. To clarify the relationship between HF and amyloid deposition, another autopsy study among those with a diagnosis of clinical HFpEF (n = 109) found that ATTR amyloid deposition was sufficient to explain the phenotype in 5% of the sample.^5^ Notably, among those diagnosed with HFpEF at an age of less than 65 years, no amyloid deposition was observed at autopsy as compared to patients with HFpEF over 80, where ATTRwt was found in 40% of patients. A more recent prospective study among 120 hospitalized older patients with HFpEF and increased ventricular wall thickness (>12 mm) on echocardiography used nuclear scintigraphy to screen for transthyretin cardiac amyloidosis, revealing diffuse myocardial retention of the radiotracer, DPD, consistent with ATTRwt cardiac amyloidosis in 13% of the cohort.^6^ In this study, the patients with ATTRwt cardiac amyloidosis were also on average older than those with other etiologies of HFpEF with a mean age of 86 vs 81 years. Importantly, an equal number of men and women were identified as having ATTRwt cardiac amyloidosis using this active ascertainment approach. This is in contrast to previously reported sex and racial differences between ATTRwt and those with hereditary transthyertin cardiac amyloidosis (ATTRh), with ATTRwt more commonly diagnosed in white men.^7^, ^8^ The results of a very recent analysis of patients hospitalized for HF again support the higher prevalence of this disease than previously recognized, delineating a prevalence of cardiac amyloidosis to be 55 per 100 000 HF hospitalizations in the United States in 2012 prior to the advent of a non‐biopsy approach to diagnosis.^9^ The definition of a rare disease in the United States is one that affects less than 200 000 patients and in Europe, is defined as one that affects less than one in every 2000 patients. This translates to a prevalence of less than 50 to 60 per 100 000 patients.^10^ Therefore, it is very likely that ATTR cardiac amyloidosis may not represent a rare disease among older patients hospitalized with HFpEF.

**Figure 1:**
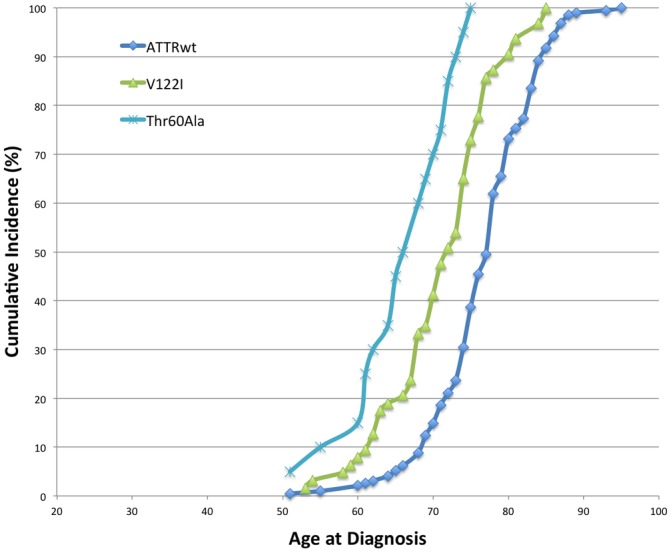
Age at diagnosis of wild‐type transthyretin cardiac amyloidosis (ATTRwt) and hereditary transthyretin cardiac amyloidosis (ATTRh) including both V122I and Thr60Ala mutations, in a single center cohort (N = 300)

It is also worth noting the age dependent penetrance of ATTRwt cardiac amyloidosis. This is important to consider when comparing to patients with ATTRh, which is an autosomal dominant disease due to mutation in TTR gene.^7^ Compared to patients with ATTRh cardiac amyloidosis, ATTRwt patients tend be older in age at diagnosis compared to patients with ATTRh, though this is dependent on the mutation in cases of hereditary ATTR. One 2012 study among a cohort of 65 patients with ATTR cardiac amyloidosis reported the average age at diagnosis of ATTRwt to be 77 vs 71 years for ATTRh with a V122I mutation, the most common ATTR mutation implicated in ATTRh in the United States.^11^ In the same, now larger cohort of 300 patients, the average age at diagnosis of ATTRwt is 77 years while the average age of diagnosis of ATTRh is younger (Figure 1). For the two most common TTR mutations in the United States, mean age at diagnosis for the V122I mutation was 71 years and for the Thr60Ala mutation was 57. Notably, there are some patients diagnosed with ATTRwt cardiac amyloidosis as early as age 47.^12^ However, in general, the age‐dependent penetrance of ATTR cardiac amyloidosis is unique compared to other etiologies of HFpEF, which tend to onset earlier as a result of hypertension, diabetes, obesity or other related risk factors. A summary table comparing ATTRwt with HFpEF is provided in Table 1.

**Table 1:** Comparison of wild‐type transthyretin cardiac amyloidosis (ATTRwt) with heart failure with preserved ejection fraction (HFpEF)

Parameter	ATTRwt	HFpEF
Age	Exclusively older adults	Predominately older adults
Sex	Predominately male	Predominately female
Cause	Amyloid deposition	Multifactorial
Blood pressure	Normal or low	High
Primary physiology	Upward shifted EDPVR	Multifactorial
Treatment to reduce mortality	TTR stabilizer—Tafamidis	None

## 3 PATHOPHYSIOLOGY

Transthyretin, which is formerly known as prealbumin, is a tetramer transport protein synthesized predominantly in the liver. With genetic mutation or with aging, this tetramer destabilizes into monomers and deposits in the myocardium, resulting in decreased left ventricular capacitance (ie, upward shifts of end diastolic pressure‐volume relation) and symptoms of HF.^13^, ^14^ Infiltration of the conduction system also leads to various arrhythmias including atrial fibrillation, bundle branch blocks, and other bradyarrhythmias. In addition to the myocardium, amyloid deposition may also occur in peripheral nerves, manifesting clinically as motor, sensory, or autonomic neuropathies. A neuropathic phenotype is more common in some patients with ATTRh depending on the particular mutation, but is relatively rare in subjects with ATTRwt cardiac amyloidosis.^7^ The gastrointestinal manifestations that occur frequently as a result of autonomic dysfunction in ATTRh are less commonly observed in patients with ATTRwt. Overall, cardiac disease is the most common manifestation of ATTRwt in the older patients affected.

**Figure 2:**
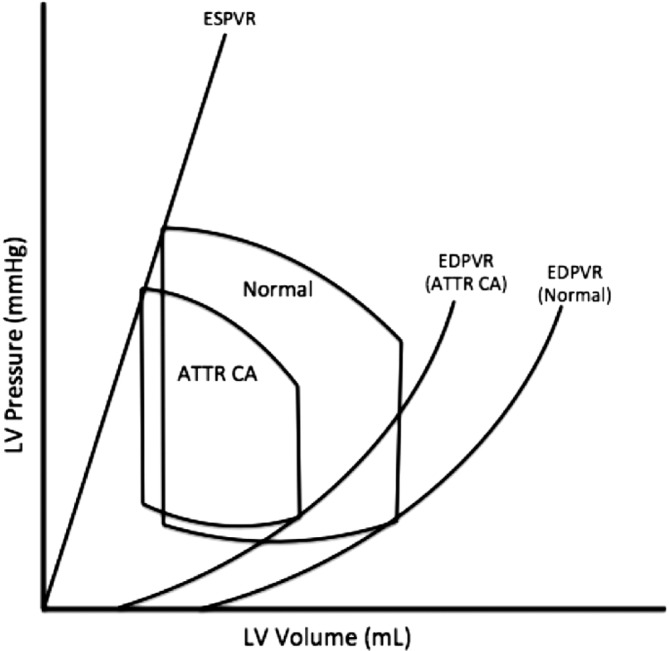
Pressure‐volume relationships in transthyretin cardiac amyloidosis (ATTR)

The diastolic dysfunction associated with ATTR cardiac amyloidosis demonstrates unique pressure‐volume alterations compared to other etiologies of HFpEF (Figure 2). With deposition of amyloid over time, ventricular capacitance declines which results in upward shifts in end diastolic pressure‐volume relationship (EDPVR) and smaller stroke volume. Additionally, higher afterload and lower contractility may play a role as the disease progresses, but decreases in ventricular capacitance are a primary feature of ATTRwt cardiac amyloidosis. The advanced features of the phenotype of ATTRwt cardiac amyloidosis seen in the ATTR‐ACT trial (low stroke volume indexes, high NT‐pro‐BNP, markedly thickened ventricular walls, positive troponins) in the context of mainly class II symptoms of HF reflects the indolent and slowly progressive process that likely begins years or even decades before the diagnosis is typically made.^15^ The described changes can be quantified with pressure‐volume analyses by reduction in end systolic elastance, a measure of chamber contractility, and increased effective arterial elastance (Ea), a measure of vascular load dependent on peripheral resistance and heart rate.^14^ As a result, ATTR cardiac amyloidosis leads to a unique HFpEF phenotype that occurs without concomitant hypertension. The diagnosis should therefore be considered in an advanced age adult with HFpEF and low or normal blood pressure or in whom antihypertensive therapy is being de‐prescribed.

## 4 DIAGNOSIS

### 4.1 Clinical clues in the older patient

The most common clinical manifestation of ATTRwt cardiac amyloidosis is HF. Symptoms of HF include dyspnea on exertion, orthopnea, and paroxysmal nocturnal dyspnea and signs of HF include crackles on lung exam, elevated jugular venous pressure, and lower extremity edema. While common with advanced disease, the HF syndrome associated with ATTRwt cardiac amyloidosis is not necessarily unique to this disease entity. Overall, the diagnosis of ATTRwt cardiac amyloidosis as the etiology of HF is challenging in part because it affects older adults who have multiple chronic conditions, many of which can mimic the symptoms of cardiac amyloidosis. However, for the prepared clinician there are several clues to the diagnosis including historical information.

There are numerous orthopedic manifestations that have been associated with presence of ATTRwt cardiac amyloidosis. Bilateral carpal tunnel syndrome in an advanced age patient is one example that should raise suspicion for systemic amyloid deposition. In one 2011 study of 100 patients with idiopathic carpal tunnel syndrome who underwent carpal tunnel release surgery, with a mean age of 67 years, 34% of surgical specimens showed TTR‐positive amyloid deposition in synovial tissue.^16^ Lumbar spinal stenosis, the most common indication for back surgery in older adults, may represent yet another potential manifestation of systemic amyloid deposition. In one study of patients who underwent surgery spinal stenosis, 21 of 26 specimens demonstrated amyloid deposition.^17^ Of the 15 specimens suitable for immunohistochemical staining, five demonstrated positive TTR immunoreactivity. Another study among 95 ligamentum flavum specimens obtained from patients who underwent surgery for lumbar spinal stenosis or disc herniation, all samples contained amyloid deposition, with 43 of 95 staining positive for transthyretin.^18^ Notably, in both studies, patients with transthyretin amyloidosis were older than those without. Biceps tendon rupture represents another orthopedic condition that has been strongly associated with the presence ATTRwt cardiac amyloidosis. In a cohort of patients (n = 111) with confirmed ATTRwt disease, biceps tendon rupture was observed in 37 patients, representing 33% of the cohort, and was a significantly more common finding in patients with TTR cardiac amyloidosis than patients with other etiologies of HFpEF.^19^ The authors of this estimate the positive predictive value of biceps tendon rupture to be 66% (LR 13.3) for the diagnosis of ATTRwt assuming a 13% prevalence of ATTRwt among those with HFpEF. Additionally, another study among 313 patients with ATTRwt cardiac amyloidosis showed a greater prevalence of total hip and knee arthroplasties in those with ATTR cardiac amyloidosis (23.3%) compared to the general population (9.2%), with arthroplasty preceding diagnosis of ATTRwt cardiac amyloidosis by ~7.2 years.^20^ Therefore, in an older patient with HF and any of these orthopedic complaints, ATTRwt cardiac amyloidosis should be considered.

There has also been an increasing literature regarding the prevalence of ATTRwt cardiac amyloidosis in older patients with various other cardiovascular disorders. In patients with aortic stenosis (AS), the presence of a low‐flow, low‐gradient aortic stenosis phenotype should raise suspicion for concomitant ATTRwt cardiac amyloidosis. A 2016 retrospective analysis studied 16 patients with AS and ATTR cardiac amyloidosis.^21^ The mean age of the sample was 79 years and 81% (n = 13) had ATTRwt. Of those with ATTRwt cardiac amyloidosis and aortic stenosis, the vast majority demonstrated both low‐flow and low‐gradient phenotype. Patients in this study with cardiac amyloidosis had an especially poor prognosis, with 44% mortality at 16 months. Further, a 2017 study used cardiac scintigraphy to screen for ATTR cardiac amyloidosis on 151 patients undergoing transcatheter aortic valve replacement (TAVR), finding 16% prevalence of ATTRwt in this cohort and 22% of men having ATTRwt cardiac amyloidosis.^22^ Again, more than half of the patients with severe, low‐flow low‐gradient AS had concomitant ATTRwt cardiac amyloidosis, supporting this association. Other retrospective studies have shown a prevalence ranging from 6% to 12% among older adult patients with aortic stenosis that have concomitant ATTRwt cardiac amyloidosis.^23^, ^24^ The association between these conditions is therefore higher than previously recognized, likely due to the age‐dependent penetrance associated with both ATTRwt cardiac amyloidosis and calcific AS. There are numerous postulated pathophysiologic mechanisms for worse outcomes associated with the presence of both conditions. For example, pressure overload induced by severe AS can exacerbate the HF syndrome associated with ATTRwt cardiac amyloidosis. Further the diastolic dysfunction associated with ATTRwt cardiac amyloidosis can lead to lower cardiac output that is already compromised by AS.^21^ It will be important to study whether or not outcomes, including those after treatment with valve replacement, are affected by the presence of concomitant amyloidosis.

### 4.2 Electrocardiographic features

Abnormalities on the 12‐lead electrocardiogram (ECG) include low voltage, conduction disease and pseudo‐infarct pattern. While findings on ECG are generally not diagnostic, they are important to consider given that ECG is one of the most common tests performed in clinical practice and therefore may be the first clue to the diagnosis in an older adult. Classically, a low voltage pattern has been considered the hallmark pattern of cardiac amyloidosis. The prevalence of this finding in patients with cardiac amyloidosis varies throughout the literature, usually according to the methodology used to define low voltage. One study of 200 patients with known cardiac amyloidosis, 55% of whom had AL amyloidosis and 45% had ATTR cardiac amyloidosis, reported the prevalence of low voltage to be 60% when using Sokolow index (sum of *R* wave in *V*_1_ and the largest of *S* wave in *V*_5_ or *V*_6_), 34% when using limb lead criteria (<0.5 mV), and 13% when using precordial lead criteria (<1 mV). The Sokolow index showed a significant association with adverse outcomes including hospitalization, transplant and death.^25^ The combination of low voltage on ECG with increased ventricular thickness on echocardiogram should raise suspicion for the diagnosis. In accordance, Quarta and colleagues studied the ratio of total QRS voltage to LV wall thickness to predict the presence of cardiac amyloidosis (ATTR and AL) among other causes of increased wall thickness, such as hypertrophic cardiomyopathy or hypertensive heart disease.^26^ In this multicenter retrospective study based on pooled data from amyloidosis referral centers, 262 patients with cardiac amyloidosis of any etiology were studied, including AL (n = 161), ATTRh (n = 71), and ATTRwt (n = 30) and controls included subjects with hypertrophic cardiomyopathy or hypertensive heart disease (n = 298). The total QRS score, the sum of voltages in the limb and precordial leads, divided by the LV wall thickness indexed to height ^2.7 had the best diagnostic performance in males (LR 3.6). In females, this method also had high diagnostic performance (LR 3.1) similar to numerous other indices. In this analysis and in others, a pseudo‐infarct pattern is another ECG feature that is more common than low voltage, seen in 70% of patients with ATTR cardiac amyloidosis, and thus more sensitive than low voltage.^25^ When the pseudo‐infarct is present in an older patient, there is the potential to misattribute the ECG findings to underlying coronary artery disease as opposed to ATTR cardiac amyloidosis. Additionally, a positive troponin, attributable to myocardial apoptosis from amyloid infiltration, may be present and can be inappropriately assumed to be the result of an acute coronary syndrome, sometimes leading to invasive assessments.^27^

Conduction abnormalities on ECG are also extremely common in cardiac amyloidosis given the infiltrative nature of the disease, though they are not specific and occur commonly with advancing age. Atrial arrhythmias, including atrial flutter and atrial fibrillation, are highly prevalent in ATTRwt cardiac amyloidosis, having a lifetime risk of 90% among patients with ATTRwt cardiac amyloidosis.^28^ Heart block and bundle branch block are also very common ECG finding in ATTRwt cardiac amyloidosis and ~30% of subjects require permanent pacing over time. A recent study of older patients with conduction disease or HF requiring pacemaker implantation screened patients for ATTR cardiac amyloidosis using nuclear scintigrahy.^29^ In this cohort (n = 143, mean age 79), there was a low prevalence of ATTRwt cardiac amyloidosis at 4% (n = 5), though only one quarter of the sample (n = 25) had HF. Larger studies are needed to clarify the prevalence among older adults with HF undergoing pacemaker implantation.

### 4.3 Noninvasive imaging

Until recently, the gold standard for diagnosis of any form cardiac amyloidosis was endomyocardial biopsy. Given that this is an invasive procedure, often only performed at specialized centers and requiring expertise not only in the performance of the procedure but in the pathological interpretation, this diagnostic modality is less than ideal for many older patients and did not facilitate early diagnosis. However, the role of noninvasive imaging has markedly expanded the diagnostic possibilities and has shown promise in its ability to diagnose the ATTR cardiac amyloidosis without the need for a biopsy.

The first and most common imaging modality that may raise suspicion for, but not diagnose cardiac amyloidosis is transthoracic echocardiography. This test is noninvasive and readily obtainable in the older adult patient. Echocardiographic findings in support of the diagnosis are well described and include biventricular wall thickening, biatrial enlargement, thickening of the interatrial septum, and the presence of a pericardial effusion.^30^ Other echocardiographic features are diastolic dysfunction and/or a “speckled” appearance of the myocardium. However, it is important to note these findings are neither sensitive nor specific for cardiac amyloidosis. Other noninvasive modalities such as cardiac MRI can raise suspicion for cardiac amyloidosis, with the characteristic finding in advanced disease being late gadolinium enhancement in a global, often transmural distribution.^31^ However, this imaging study is expensive and requires contrast, which can be problematic in the older adult with renal dysfunction. Importantly, a recent prospective study of 868 patients with suspected cardiac amyloidosis validated the use of T1 mapping without the use of gadolinium, which may expand the role for MRI in diagnosis.^32^

**Figure 3:**
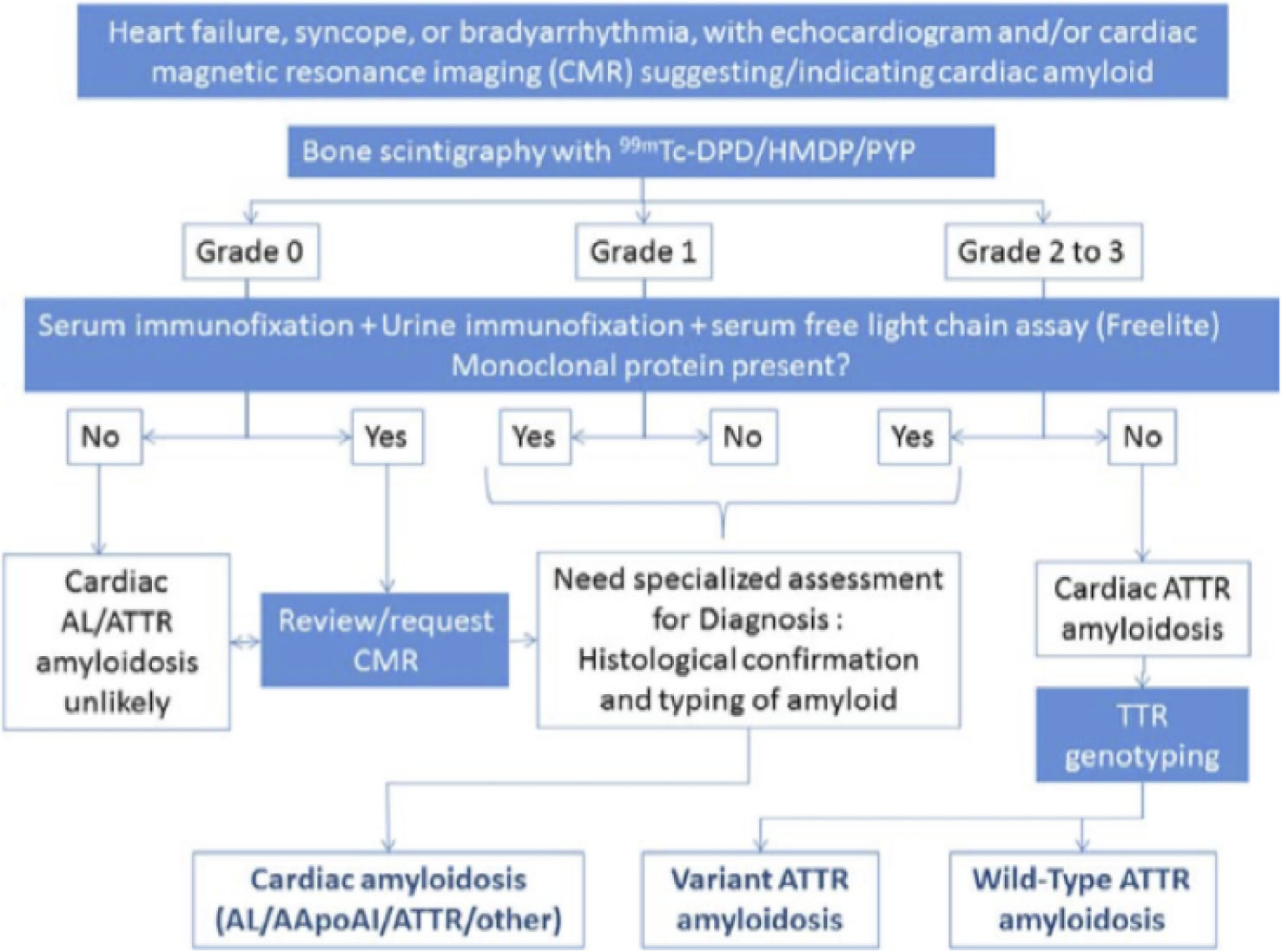
Diagnostic algorithm for patients with suspected transthyretin cardiac amyloidosis (ATTR). Reproduced with permission from Reference Gillmore et al^35^

Nuclear scintigraphy is the noninvasive imaging modality that can establish the diagnosis of ATTR cardiac amyloidosis without the need for biopsy. Scintigraphy uses technetium‐labeled bisphosphonates to localize amyloid deposition to the heart. Cardiac retention of these radiotracer is scored according to a grading system devised by Perugini et al with grade 0 indicating absent myocardial uptake, grade 1 indicating mild myocardial uptake less than bone, grade 2 indicating moderate myocardial uptake equal to bone, and grade 3 indicating high myocardial uptake greater than bone.^33^ Importantly, this modality may identify amyloid deposition before the development of abnormalities on echocardiography or MRI, leading to earlier diagnosis.^34^ The consensus for use of scintigraphy as a non‐biopsy approach to diagnosis comes from a large cohort study of 1217 patients with suspected cardiac amyloidosis of which 374 had endomyocardial biopsy and all of whom underwent nuclear scintigraphy.^35^ For these cases, radiotracer uptake was found to have over 99% sensitivity and 86% specificity for diagnosing ATTR cardiac amyloidosis. Lower specificity in this study resulted from a false positive scan that can occur in cases of AL amyloidosis. However, in the setting of negative serum and urine immunofixation and normal serum free light chain ratio, the positive predictive value for cardiac ATTR amyloid with grade 2 or 3 uptake on nuclear scan was 100%. A subsequent multicenter analysis of 171 patients, 121 with TTR cardiac amyloidosis, not only confirmed the high sensitivity and specificity of scintigraphy but also showed a higher degree of uptake to be associated with worse survival.^36^ There are other scintigraphic features used to differentiate AL vs ATTR cardiac amyloidosis. A 2014 study of 45 subjects with cardiac amyloidosis (73% ATTR cardiac amyloidosis, 27% AL amyloid) assessed cardiac retention of radiotracer by calculation of a heart‐to‐contralateral ratio (H/CL) in which a region of interest is drawn over the heart and corrected for contralateral counts.^37^ A H/CL ratio over 1.5 was 97% sensitive and 100% specific for ATTR cardiac amyloidosis. Of note, this ratio and cutoff for diagnosis depends on the time of assessment as the isotope washes out of the myocardium over time. In accordance with the above findings, a diagnostic algorithm has been previously published to facilitate early and accurate diagnosis in the older patient who is suspected to have cardiac amyloidosis (Figure 3).^35^

## 5 TREATMENT

### 5.1 Traditional medical management

Until recently, the options for medical therapy of ATTRwt cardiac amyloidosis were markedly limited. The mainstay of the medical management of ATTRwt cardiac amyloidosis is maintenance of euvolemia with diuresis. Given that the population affected is older with multiple medical comorbidities often including renal dysfunction and ventricular vascular stiffening, it is a challenge to maintain euvolemia in this population. Neurohormonal blockade (ie, angiotension‐converting enzyme inhibitors and angiotensin receptor blockers) that has clearly demonstrated mortality benefit in patients with HF and reduced EF (HFrEF) has not shown the same benefit and may actually worsen outcomes in those with cardiac amyloidosis. These medications may induce clinically significant hypotension given that the autonomic neuropathy associated with cardiac amyloidosis may lead to orthostatic hypotension at baseline.^38^ Additionally, beta‐blockers and calcium channel blockers have been observed to be harmful in patients with cardiac amyloidosis and are often poorly tolerated.^38^, ^39^ In fact, a HF syndrome that worsens with administration of these medications can also serve as a clue to the diagnosis. Due to the fixed, small stroke volume associated with cardiac amyloidosis, lowering heart rate can compromise cardiac output.

### 5.2 Stabilizers, silencers, and degraders

With the emergence of targeted therapies to treat the specific pathophysiology associated with disease, the designation of ATTR cardiac amyloidosis as a universally fatally disease is now challenged. At this point in time, transthyretin stabilizers, which work by preventing tetramer destabilization, the rate limiting step in transthyretin amyloid production, have been studied most extensively.^40^ Diflunisal and tafamidis are two transthyretin stabilizers that first demonstrated safety and effectiveness in the treatment of familial amyloid polyneuropathy (FAP).^41^, ^42^, ^43^, ^44^ For ATTR cardiac amyloidosis, the data for diflunisal is limited. One 2012 study examined the safety and efficiency of the drug in a small cohort of 13 patients with TTR cardiac amyloidosis, concluding overall safety of administration however noted a 6% decline in EGFR with administration, which may be problematic in an older age population with renal dysfunction at baseline. Another single center retrospective cohort study of 13 patients receiving diflunisal for ATTR cardiac amyloidosis found lower risk of a combined outcome of death or transplant in these patients.^45^ Overall, diflunisal is inexpensive and may be safely given to those with early, stable disease, and preserved renal function who are not on high dose diuretics.

Tafamidis has demonstrated efficacy in the treatment of TTR cardiac amyloidosis. In 2019, a large phase III randomized, multicenter trial studied the use of tafamidis in 441 patients with TTR cardiac amyloidosis who had a mean age of 74 years.^15^ This trial found lower all‐cause mortality at 30 months in patients treated with tafamidis compared to placebo. Treatment was also associated with fewer hospitalizations and improved functional and quality of life outcomes, which is an important finding in this older age cohort. Additionally, there were no significant side effects compared to placebo, which is important in the context of multimorbidity. Notably, this trial excluded patients with NYHA class IV HF and those with significant renal disease (EGFR < 25) and therefore this treatment is not indicated for these patient subgroups. This trial established tafamidis as an effective medical therapy for the disease, which was approved by the FDA in May 2019, albeit at cost that will likely impact widespread application. Another stabilizer currently under investigation is AG10, which has shown safety and efficacy in a phase II study^46^ and is enrolling subjects in a phase III registration trial. AG10 binding to TTR mimics the T119M rescue mutation making it a potent and promising candidate for the treatment for ATTR cardiac amyloidosis.^47^

Other methods of treatment have shown efficacy in ATTRh polyneuropathy and are currently under investigation for ATTR cardiac amyloidosis. These treatment strategies include suppression of transthyretin synthesis with small interfering RNA and antisense oligonucleotides.^48^ Patisiran is a small inferring RNA that has been studied in phase III multicenter controlled trials in patients with ATTR hereditary polyneuropathy. In those with cardiac involvement (n = 126, 56%), patients randomized to treatment with pasitiran showed preserved cardiac output compared to placebo and other indications of less ventricular strain including higher left ventricular end‐diastolic volume and lower LV wall thickness and mass.^49^ Inotersen is an antisense oligonucleotide drug that inhibits hepatic production of TTR amyloid that has been studied in a phase III trial in patients with hereditary FAP, the majority of whom demonstrated a cardiac phenotype (n = 108, 63%). Administration of the drug in a sample of 172 patients led to improvements in the primary outcome of functional capacity and quality of life assessment though no difference in ventricular strain over time.^50^ Both approaches to TTR silencing are being applied to ATTRwt cardiac amyloidosis, with phase III trials anticipated in late 2019 and early 2020.

### 5.3 Future directions

ATTRwt cardiac amyloidosis is a disease with an age‐dependent penetrance that exclusively affects older adults. ATTRwt cardiac amyloidosis was historically difficult to diagnose and to treat, leading to universally poor quality of life and survival. However, for a prepared provider, there are numerous well‐defined clinical and imaging characteristics that can facilitate earlier diagnosis. At the same time, there are now effective treatments, and ideally more being tested in late phase clinical trials. Overall, the marked advancement in the diagnosis and treatment of ATTRwt cardiac amyloidosis can be attributed to a better understanding of the precise pathophysiology of the disease and applying therapies targeted to these mechanisms. Experience in ATTRwt cardiac amyloidosis can serve as a model to advance the study of HFpEF in general.
